# Room for interpersonal relationships in online educational spaces – a philosophical discussion

**DOI:** 10.1080/17482631.2019.1689603

**Published:** 2020-10-25

**Authors:** Catrine Kostenius, Eva Alerby

**Affiliations:** a Department of Health Sciences, Luleå University of Technology, Luleå, Sweden; b Department of Art, Communication and Education, Luleå University of Technology, Luleå, Sweden

**Keywords:** Health, Well-being, Interpersonal relationships, Online education, Paradigm case, Philosophical

## Abstract

**Purpose:** To explore interpersonal relationships within online educational spaces and to connect the discussion to health and well-being among students and teachers. **Method:** We apply different perspectives to analyse the complexity of interpersonal relationships in online educational spaces, based on the philosophies of Nel Nodding, Maurice Merleau-Ponty, and Alfred Schutz. We use a qualitative methodological combination—philosophical explorations, literature review, and text analysis—to offer significant insights that will substantially inform contemporary theories in research addressing interpersonal relationships in online education. **Results:** We illuminate and theorize about interpersonal relationships in terms of being cared for, confirmed by, and connected to others as dimensions of significance for health and well-being in educational spaces in the form of flexible online courses at the university level. **Conclusion:** We argue that all education should strive to provide caring relationships and educate for both self-understanding and group understanding, and contribute to school as a place where happiness and joy for genuine learning and knowledge are promoted. To achieve this, it is of significance to also be connected to and confirmed by others, teachers as well as students, and also to exist in a (class)room that provides room for interpersonal relationships: in real life or online.

## Introduction

In the 21st century, there are constant transformations of knowledge and information, based on the rapid technological, economic, social and educational changes. This, in turn, can be termed “the era of the digital age”, and presents new opportunities that transform people’s lives in an intense way (Reichenberger, 2017). Research so far has, however, not paid much attention to the role of digitalization in identity formation and interpersonal relationships (Atay, 2015). In the era of digitalization, formal education more often takes place as flexible online courses that might affect human beings in multiple ways. This, in turn, calls for an exploration of the complexity of educational spaces (Alerby, Arndt, & Westman, 2017), especially in relations to interpersonal relationships. To achieve knowledge, interpersonal relationships are of significance for not only learning but for human beings’ health and well-being (Bragg, 2007).

There are some arguments for interactive technology in the educational spaces for learning, which can either help or hinder the promotion of health and well-being (Kostenius, Bergmark, & Hertting, 2017). Among researchers, there are different ideas about the use of interactive technology; some stress the negative health aspects, such as having low levels of connectedness in relationships (Laura & Chapman, 2009). According to Toufaily, Zalan and Lee (2018), students in online education experience belonging and social interactions sacrifices, related to loss of human touch and real-time interactions with teachers and peers: “you miss this feeling of human presence” (p.31). House-Peters, Del Casino and Brooks (2019) are deeply troubled by the offloading of responsibility for internet access, speed, and associated costs to the individual student. However, they also note the positive aspects of online education, such as a potential for allowing new voices, aligning well with education approaches that enable participants that otherwise might not have access to educational programmes. Others have also argued for the opportunities of online communication as they found that it enhances self-esteem, relationship forming, friendships, and increases friendship quality (Valkenburg & Peter, 2011). According to students, the use of interactive technology in school promotes good relationships as it offers a way to socialize, provides a tool for meeting, and gives support when encouraging classmates (Kostenius & Hertting, 2016). Also, students’ experiences of health and wellbeing in the school context point to the importance of being cared for, confirmed by and connected to others (Kostenius et al., 2017). Therefore, spaces for education need to facilitate not only the achievement of academic goals but also the fundamental needs of interpersonal relationships (Kostenius & Hertting, 2016).

This paper aims to explore interpersonal relationships within online educational spaces and to connect the discussion to health and well-being among students and teachers. We apply different perspectives to analyse the complexity of interpersonal relationships in online educational spaces, based on the philosophies of Nel Nodding, Maurice Merleau-Ponty, and Alfred Schutz.

Nodding’s (2005) theory supports the exploration of ethical dimensions of interpersonal relationships, while Merleau-Ponty’s life-world approach (Merleau-Ponty, 2002; Merleau-Ponty & Lefort, 1968)–with the addition of experienced social reality suggested by Schutz (2002)–supports a re-imagination of educational spaces. We will illuminate and theorize about interpersonal relationships in terms of being cared for, confirmed by, and connected to others as dimensions of significance for health and well-being in educational spaces in the form of flexible online courses at the university level. Also, we hold an ontological discussion of what an educational space, in the form of a (class)room, *is* and *can be*, and in what way the space might interplay with interpersonal relationships. We use a qualitative methodological combination–philosophical explorations, literature review, and text analysis–to offer significant insights that will substantially inform contemporary theories in research addressing interpersonal relationships in online education. This is in response to urgent calls for increasingly philosophical engagement in education (Koro-Ljungberg, Löytönen, & Tesar, 2017; Peters, 2007; White & Peters, 2011). The paper should, therefore, be viewed as a theoretical contribution to the field of health and well-being in a broad sense—more specifically, the significance of interpersonal relationships in online educational spaces. The exploration is exemplified by, and connected to, some university students’ and teachers’ experiences through a narrative in the form of a paradigm case. The paradigm case is constructed by us as researchers, based on our own, colleagues and students previous experiences of online education and interpersonal relationships (cf. Alerby & Hertting, 2011; Alerby & Hörnqvist, 2003).

## Interpersonal relationships within online education—a paradigm case

The point of departure for our exploration is in an online (class)room at a university programme in a university in Sweden. The scene of the story is taken from a lesson in health promotion and is based on conversations with teachers and students about teaching and studying within a program that is mainly taught through online education. There are many different kinds of online educational environments, with a number of multi-dimensional interactional spaces using several technologies for different aspects of the pedagogical journey, for example pre-recorded video lectures and assignments, chat rooms and peer online discussion forums for students to interact asynchronously. However, in this paradigm case, the example of the online education is situated in a synchronous online space. This means that the students and the teacher are meeting at the same time and in the same online (class)room. In other words, the persons participating in the lesson can be logging in from different geographical locations, but synchronously. The example is chosen due to the focus on interpersonal relationships, and how these relationships might interplay and affect health and well-being among teachers and students. Further, the example chosen for our analysis involves a teacher who is fairly unfamiliar with synchronous online educational technologies and the various pedagogical tools available in such environment. The story begins with a lesson in which a group examination is taking place.

There are 18 of 24 students signed in to the online (class)room where David, the head teacher in the course, is waiting with his headset on and a welcoming smile—displayed on the frozen picture. David is an experienced teacher in the subject of health promotion. However, the online format of teaching started only 3 months ago, when the educational program he worked at changed from education in regular old-fashion classrooms on campus, to online education off campus. David is a bit nervous about the technical aspects of the lesson but wants to take charge of the two upcoming 45-min sessions. The focus of the day is a group examination of a course assignment, so David tries to give clear explanations of what to do:

1. Hello everyone, welcome to class today, please turn on your camera so I can see your face and say hello as I read your name. Then, turn off your cameras afterwards. Anna M!
2. Hello, I’m here.
3. George!
4. Yes (waving and smiling).
5. Amanda?
6. I’m here (no picture).
7. I can’t see you.
8. No, I know, it’s something wrong with my camera.
9. You know it’s a prerequisite to being able to participate in the examination to be online with both microphone and camera, so I can see you and hear you.
10. I know, but I can’t get my camera to work.
11. Well, then I don’t know what to do.

Monica, one student in the group, pushes the button for “raised hand”, but David does not notice this. She then decides to join the conversation anyhow to help the teacher and the classmate to solve the problem. She turns on her microphone and suggests:

1. Amanda, you can maybe log in with your mobile?
2. Okay, I’ll try to do that, I’ll be back.
3. Thank you, Monica, for the suggestion. Let’s see if it works. Next is Mats! (no one answering) Mats?
4. I think he has a dentist appointment or something like that.
5. Okay lets, move on. Jessica?
6. Good morning from Uppsala.

David goes through the list of students, and meanwhile, two more students log on, so now there are 20 students signed in for the group examination. Amanda is not eligible as she has no camera turned on, which is a prerequisite to participate. She tried to connect via her mobile phone but was unable. In an attempt to solve the technical problem David decides that she can partake in the examination together with her group and then get an extra assignment to fulfill the requirement. David was not entirely happy with the solution, but Amanda agreed, and the problem was solved for the moment. Then, the examination begins with instructions from David about the format, and he underlines that the presenting students need to have their cameras on. The first group switches on their cameras and make their presentation. David, who has been silent for 10 min making notes while listening to the presentation, turns on his microphone.

1. Well done group one! I see that five of your classmates pushed the applause-button, nice. And now any comments from you who have listened?

Three students push the button for “raised hand” and David notes this.

1. I see that three of you have a comment, Klara you can start.
2. You said something about promotion and prevention being the same thing, what did you mean?

It’s silent a few seconds, and David jumps in.

1. Group one, who would like to answer?

Olle turns on his speaker and explains:

1. Well, let’s say you are keeping fit by being physically active, then you are strengthening your health, that’s health promotion, but you can also look at the physical activity as disease prevention because you are less likely to become overweight or suffer from diabetes and heart disease.
2. Okay, I get you now.

David gives positive feedback and moves the session long.

1. Thank you, Olle, a very nice explanation. And now Sebastian you raised your hand, what did you want to ask?
2. Maria, you said that Antonovsky’s model came from studies about battered women, that’s not right.

Maria turns on her speaker and says, notably irritated:

1. Yes, I have read that in an article.
2. But that’s not right because in our course literature it says that it was with women from concentration camps.

David clears his throat:

1. Sorry Maria but Sebastian is right, you are incorrect. You see, Aaron Antonovsky is known for his SOC model, SOC stands for sense of coherence. As explained in the course literature the origin of the salutogenic idea was in one of his studies on Israeli women, some of whom had been in concentration camps. Antonovsky recognized that there were women who had suffered greatly in life, for example by sitting in concentrations camps, and despite this reported that they were in good health. He then posed the salutogenic question something like this: how is it possible that some people, despite terrible experiences, experience good health?

Maria turns off her camera and a few seconds later she is offline. David tries to make contact.

1. Maria, are you there?

Uncomfortable silence in the online (class)room.

How can this narrative, in the form of a paradigm case, be understood? What happens when the horizon of the (class)room becomes infinite, and the teacher and the students are located at different physical and geographical places? In what way are interpersonal relationships intertwined and affected by this situation? Let us analyse and discuss this by turning to the work Maurice Merleau-Ponty by starting to elaborate on what a (class)room is and can be—the setting where interpersonal relationships take place.

## Some notions of what a (class)room is or can be

Probably no one questions that a university building has classrooms—instead, this is often taken for granted. But taking something for granted can be a reason to return to the thing itself, in this case, “the classroom” as a space. Merleau-Ponty reminds us that we need to interrogate our presumptions through what he describes as hyper-reflection, a critical self-reflection that interrogates its own possibility (Merleau-Ponty & Lefort, 1968). A question that arises is how a classroom can be understood and explained. To elaborate on and discuss what a (class)room is and can be is of significance for our further exploration of interpersonal relationships within online educational spaces.

At an ontological level, there are different answers to this question, and there are several ways to understand and explain what a classroom is and can be. A classroom can be understood and explained based on its material properties–from a materialistic viewpoint. It can also be viewed from an idealistic point of view–a (class)room can be understood and explained as an idea. However, in addition to the materialistic and idealistic point of views, other perspectives are also found in the room’s utility quality–the room can be used for something by someone. Thus, the characteristics of the room cannot be reduced to only those found in the material or ideological perspectives. Instead, the utility quality of the room adds a further dimension to the understanding of the room, which additionally requires a subject who experiences the room. Things are always things for someone and the room, for example, requires someone who experiences it (Alerby, Hagström, & Westman, 2014). The same is true for the online (class)room—it can be described in the form of material properties or an idea, but also for its utility quality.

Even though there are similarities between a physical classroom and an online (class)room, there are also differences, including the shifting horizons of the rooms. One way to understand the horizon of the room is to say that it begins and ends at the walls of the room (even though there are classrooms without any walls). In an ordinary physical classroom, the boundaries of the room most often consists of four walls, a ceiling, and a floor, and ways to stretch the horizon of the room can be to look out of a window or walk out the door. Another kind of stretching the horizon of the (class)room happens in connection with online education. In these contexts, the teaching extends through the ether to completely different places and spaces than the ordinary physical classroom. In this way, it can be said that the (class)room’s horizon is shifted to infinity and learning takes place where the person in question is presently located. Regardless of how the room is viewed, as a physical classroom or as an online (class)room, the room can be considered as a kind of framework for the actions of the teacher and students, and this framework is of significance for the interpersonal relationships within the room. Let us, therefore, further elaborate on this by continuing to use the philosophy of Maurice Merleau-Ponty, and adding the work of Nel Nodding, and Alfred Schutz.

## Philosophical explorations of interpersonal relationships in the (class)room

According to Merleau-Ponty (1996), there is a mutual interplay between human beings and the world. Furthermore, he claims that it is through the body that a person is in a living relation to things, such as a laptop or a headset. Expressed in other words, the world (e.g., new media technologies) influence human beings, and vice versa. Even though the phenomenon of new media is, as the word indicates, relatively new, it has been used in an educational setting for quite some time, and online education constantly increases. There are several benefits to this kind of education. Flexible online courses can, for example, create opportunities for students in remote areas or whose lifestyle does not fit with physically attending classes. However, when something is “new”, it is a learning process for all involved, teachers as well as students, to get used to, and achieve knowledge of the new technology and methodology of teaching and learning. For the knowledge to be established, or embodied as Merleau-Ponty (1996) expressed it, a habit must be formed. When the teacher and the students within an online course are having a lesson for the first time, they are probably, to some extent, more or less unsure about the technical solutions. This is evident in the paradigm case:

“the online format of teaching started only three months ago when the educational program he (David, the teacher) work changed from regular on-campus education in regular old-fashion classrooms on campus, to online education off campus. He is a bit nervous for the technical aspects.”

When the online format of the educational situation is new, it is a distance, not only between the humans located at different geographical places but also between the humans and the technique, for example in the form of the computer, the screen, and the headset. To really have the knowledge embodied, which means that a habit is formed, the equipment, the technique, and the methodology has to become one with the humans lived body (Merleau-Ponty, 1996). It is quite obvious that the teacher David does not yet have the online teaching in his body. Furthermore, another example of this is that he does not notice when the student is trying to get his attention by using the button for “raised hand”: “Monica, one student in the group, push the button for ‘raised hand’, but David does not notice this”. One way to analyse and understand this fact is that a habit to look for signs from the students has not yet been formed.

It is also evident that some of the students, like Amanda, have not yet formed a habit of using the technology and have not yet embodied it, while some others, like Monica, are more familiar with it. Monica offering her assistance to Amanda can also be seen as Amanda being cared for. This can be compared to Noddings (2005) notion of caring in education, as she argues that caring should be the main aim of educators and be encouraged between peers. She concludes that “the living other is more important than any theory … a central idea in an ethic of care” (p.xix). In connection to this, the question concerning *who is teaching who* arises. Is it always the teacher who has the knowledge about everything, or are the students sometimes more skilled and have more knowledge than the teacher?

In the paradigm case, it seems that Monica is more skilled with the technology of online education and in connection to this has more knowledge than David, the teacher. A crucial aspect, as discussed by Alerby and Hertting (2011), is how the teacher is reacting to this fact–whether the teacher is viewing the student’s knowledge and skills as a threat or a resource. If this evokes negative feelings for the teacher, there might be a risk of a power struggle as the teacher experiences loss of power. This can be compared with Menon’s (1999) description of being psychologically empowered as “a cognitive state characterized by a sense of perceived control, competence, and goal internalization” (p. 162). If the teacher feels he is less competent and, therefore, dis-empowered, the relationship with the student might be negatively affected, depending on how the teacher handles this moment of *the student teaching the teacher*. According to Noddings (2005), the student-teacher relationship is unequal, but their relationship is marked by reciprocity. Furthermore, she argues that the students cannot be expected to teach the teacher, yet are expected to respond sensitively. When these moments of flipped teacher-student roles occur, there is indeed so that the student is teaching the teacher. This can be compared with Noddings (2005) discussion about teacher training, what teachers need to be well prepared and to be the best teacher they can become. She argues, for example, that teachers should be able to discuss matters on which they have no specific training “ … the worship of expertise must go … in its place we should strive for for a superbly well-trained capacity for inquiry … ” (p.178). Due to the rapid development of using technology in education, exemplified in the paradigm case, there is a need to re-negotiate teacher-student roles, where student participation and the student’s voice can be recognized along the line of Noddings take on the preparation of teachers.

However, there is always a power imbalance between the teacher and students, as well as between humans and the place. It is, therefore, of significance to emphasize that whether the context of the place is a physical classroom or digital (class)room, the place is always embraced by different kinds of boundaries, visible or invisible to the eye. When stretching the boundaries of the classroom the human relationships within the place are affected. In an ordinary physical classroom, the students can be controlled or restrained bodily by the room and the teacher (Alerby et al., 2014). In the online (class)room, the students are, to some extent, also controlled by the boundaries of the room and the teacher. The persons in the room are not bodily connected, so instead of raising their hands physically to get attention and get in touch with the others, they have to push the button “raised hand” before speaking. Another example, shown in the paradigm case is that the camera has to be switched on, or off, in accordance with the teacher’s instructions. The students can also, like Maria did, switch off the camera, in this case, due to a disagreement in connection to the reading of the course literature. Noddings (2005) discusses the need for rules in a classroom, and in connection to Maria’s disappearance, it is worth discussing the ethical approach in the online (class)room.

Using new technology, such as the online education technique exemplified in the paradigm case, entails that new forms of interpersonal relationships might occur, as discussed above in connection to knowledge and power. Another aspect is that neither the teacher nor the students are in the same room with their bodies. Instead, they can just see each other’s faces through the screen, but only when they are asked to turn on their cameras at the beginning of the lesson: “Hello everyone, welcome to class today, please turn on your camera so I can see your face and say hello as I read your name. Then turn off your cameras afterwards”, or when they are examined: “You know it’s a prerequisite to being online with both microphone and camera, so I can see you and hear you, to be able to participate in the examination”. Given this it is obvious that online education is separating the participants’ bodies–they do not exist with their bodies in the same physical room. Schutz (2002) emphasized that the world of directly experienced social reality is where “I” and “you” become “we”, it is kind of bodily coexistence in space and time. In the example shown in the paradigm case, the teacher and the students do not *bodily* coexist in space and time, nor do the students with each other. However, they do coexist in the online space and also in time–they are in a way alone together in the same space, at the same time. A question to consider is in what sense the filter of the screen, and the fact that both the teacher and the students are at different places and meet each other only through the ether, affects the interpersonal relationships. This fact might run the risk of the distance leading to indirect relations, where the use of ideal types is to compose an understanding of each other–to the world of contemporaries, as Schutz (2002) expressed it. In connection to Schutz’s argumentation about ideal types, it is worth considering whether Maria, who just left the (class)room after being reprimanded and corrected by both the teacher and a classmate—“Maria, you said that Antonovsky’s model came from studies about battered women, that’s not right […] in our course literature it says that it was with women from concentrations camps. […] Sorry Maria but Sebastian is right, so you are incorrect”—runs the risk of being viewed as a person that always leaves, instead of staying and continue the discussion: “Maria turns off her camera and a few seconds later she is offline. David tries to make contact. Maria, are you there?”.

The way the teacher reprimanded and corrected Maria in front of the rest of the class is polar opposite of Noddings (2005) description of a caring teacher, as she explains that as teachers “we do not tell our students to care, we show them how to care by creating caring relations with them” (p.22). Siegel (1999, 2012)) concludes that people feel that when they are being listened to they are “feeling felt”. Furthermore, Noddings (2005) explains that an open dialogue is important to establish a caring relationship as “it connects us and … provides us with the knowledge of each other that forms a foundation for response in caring” (p.21). The response according to Noddings (2005) is, for example, about confirming to build caring relationships. The teacher gave the students confirmation: “Well done group one! I see that five of your classmates pushed the applause-button, nice” and when David (the teacher) gives positive feedback and moves the session along, “Thank you, Olle, a very nice explanation”. In these cases, the setup of the online (class)room enabled caring relationships as being positively acknowledged is an act of showing that the other is valued (Kostenius, 2008) and “confirmation lifts us toward our vision of a better self” (Noddings, 2005, p. 25). Another example of being cared for is when David, the teacher, adapt the situation to solve the technical problems that occurred with Amanda’s camera: “In an attempt to solve the technical problem David decides that she can partake in the examination together with her group and then get an extra assignment in order to fulfill the requirement”.

The ethical dimensions of interpersonal relationships are other aspects of Marias reaction of being reprimanded and corrected worth elaborating on. These are clearly shown when Maria turns off her camera and goes offline, even though the lesson is in progress. This can be argued for as Maria having the power to remove herself from a situation in which she was not comfortable, increasing autonomy. Similarly, Kostenius and Hertting (2016) conclude that students express that interactive technology gives them a sense of control. They go on explaining that meeting and learning online, or in a virtual space, offer students opportunities to take responsibility and exercise self-control. In the case of Maria, her self-control might be in question. However, the format of the online (class)room made her in charge of her participating or not. Another interpretation is that Maria leaving the online (class)room was a form of protest against being questioned by a classmate, which was enforced by the teacher concluding that she was incorrect. Noddings (2005) argues, for the sake of caring relationships between teacher and students, that a “good teacher does not reject what students see and feel, but rather, work with what is presently seen and felt to build a stronger position for each student” (p.107). The teacher who said that Maria was incorrect, thereby rejecting her comment, did not align with Noddings view of a good teacher nor that of a caring relationship. Noddings’s (2005) notion of reciprocity, where both teacher and student have a responsibility to meet the other in an understanding way, was not upheld as Maria’s exit made it impossible to continue the conversation, leaving the situation unresolved. The online (class)room made this moment of uncaring exchange possible by its virtual nature, where one can leave in a blink of an eye, which is not possible in a traditional classroom.

Another issue worth considering is that, following Maria’s exit of the room, a silent space occurs after she ended the session: “Uncomfortable silence in the online (class)room”. Bateson (1987) states that not only spoken words, but also silence, can convey a message—the silence is saying something. Given this, Maria’s silence, followed by her absence, has a message. The question is, however, what the message is, and several interpretations follow. According to Merleau-Ponty (1996), every person has a silent and implicit language, that goes beyond the spoken language. This is something more and something different than just to avoid spoken words. Maria’s silence, followed by her exit, can be viewed as a conscious act, but according to Merleau-Ponty, to be viewed as a silent person, the person in question must have the ability to actually express something verbally, but for some reason the person chooses to be silent, which is what Maria does. Maria can then, according to Merleau-Ponty, only be viewed as silent if she has something to add to the discussion, but choose not to—she remains silent by her absence.

## Concluding remarks on the interweaving between interpersonal relationship and health and well-being

As stressed at the beginning of this paper, nowadays, formal education more often takes place beyond the physical classroom, as flexible online or web-based courses. This can be viewed as an attempt to make education more accessible, with the ultimate goal of competing in the contemporary global marketplace. This relatively new phenomenon places great demands on both the teacher and the students. As described in the paradigm case, the teacher was lacking online teaching experiences. Even though, there are an increasing number of university teachers with extensive experiences, nevertheless, the situation in the paradigm case in not uncommon—not all teachers (nor university students) are digital natives (c.f. Prensky, 2001). An issue to further explore is whether the situation in the paradigm case had been different if the teacher had been more experienced concerning online education. Teachers experiences, knowledge and skills are of significance for any educational situation and the communication that takes place with the students. To be the best teacher one can be is, according to Noddings (2005), about being well prepared. The educational situation including communication (or miscommunication), affecting both wellbeing and learning, does not only apply to online education, but can be seen in face-to face educational contexts as well (c.f. Alerby & Hertting, 2011).

However, to elaborate on the consequences of this (new) teaching form, we have turned to Merleau-Ponty (1996), who claims that establishment of knowledge–to have the knowledge embodied–is dependent on a habit having been formed. Schutz (2002) emphasizes the importance of bodily coexisting in space and time, for “I” and “you” to become “we”. A noteworthy dimension in online educational settings is therefore the lack of bodily presence and whether this affects the experiences of well-being and learning. Additionally, a question that arises is, in what way are interpersonal relationships affected if the teacher and the students do not bodily coexist in space and time during a lesson, but also in what way this might, or might not, influence and affect health and wellbeing among students and teachers.

The philosophical exploration of interpersonal relationships within online educational spaces, in connection to health and well-being among students and teachers, can be understood as a glass half filled with water. As commonly used as a way to describe a person’s perspective as being negative—viewing the glass half empty, or being positive—viewing the glass half full. We argue that in the case of interpersonal relationships in the online (class)room both perspectives are relevant, just as in real life encounters. However, there is a need for an ethical discussion illuminating the pros and especially the cons with meeting online. There are barriers for health and wellbeing, for example, not looking each other in the eye when speaking, not fully connecting, not being able to read body language since there is no view of the body or communicating via symbols instead of with your body “raising hand” or “applauding” can be seen as not sharing the same reality. One can recognize the risk of low levels of connectedness in relationships, which according to Laura and Chapman (2009) can affect health and wellbeing negatively. Furthermore, the “feeling felt” aspect when truly being listened to and understood, described by Siegel (1999, 2012) as of great importance in human relations, can be harder to realize. However, the paradigm case also exemplifies opportunities to promote health and wellbeing—the glass half full—for example lifting others via symbols like “applauding” or being able to have control over participating or not, thereby increasing autonomy. In addition, not having to show your body or face when speaking, thus putting less emphasis on the body. These examples can be compared to the arguments made by Kostenius, Bergmark and Hertting (2017) and Valkenburg and Peter (2011), that the use of interactive technology enhances self-esteem, promotes good relationships, and provide a tool for meeting and give support when encouraging others. In other words, the online meeting also enables what Noddings (2005) describes as being confirmed and cared for to build caring relationships. Also, the sense of control that is exemplified in the paradigm case can be compared to empowerment as a process focusing on human rights and the capacity to actively participate in and influence our own lives (Melander-Wikman, 2007). Empowerment is a concept found in *the Ottawa Charter for Health Promotion* (1986) and elaborated on in recommendations by the World Health Organization (1998), stating that initiatives should enable individuals to assume more power over factors that affect their health.

Online (class)rooms emanate from and consist of other conditions than regular physical classrooms. Instead of, for example, viewing a classroom as consisting of walls, floor, and ceiling, the room can be extended through the ether to completely different places and spaces. It can then be said, as emphazised above, that the horizon of the classroom has shifted to infinite and learning takes place where the person in question is at the moment. This is a kind of horizon displacement of the classroom, which occurs in connection with online education. Nevertheless, it is a (class)room, and it has a profound influence on interpersonal relationships within the room, and by that also on the health and wellbeing among the persons in the space. It is a kind of social reality, to use Schutz (2002) terminology, that supports a re-imagination of educational spaces, beyond the regular and physical. Turning once more to Noddings (2003, 2005), we argue that all education should strive to provide caring relationships and educate for both self-understanding and group understanding, and contribute to school as a place where happiness and joy for genuine learning and knowledge are promoted. To achieve this, it is of significance to also be connected to and confirmed by others, teachers as well as students, and also to exist in a (class)room that provides room for interpersonal relationships: in real life or online.
